# A randomised dose-response study of prophylactic Methoxamine infusion for preventing spinal-induced hypotension during Cesarean delivery

**DOI:** 10.1186/s12871-020-01119-2

**Published:** 2020-08-12

**Authors:** Feng Fu, Yu-wen Tang, Hong Chen, Cui-cui Jiao, Na Ma, Xin-zhong Chen

**Affiliations:** Department of Anesthesia, Women’s Hospital, Zhejiang University School of Medicine, Xueshi Rd 1#, Hangzhou, China

**Keywords:** Cesarean delivery, Hypotension, Spinal anesthesia, Dose-response, Vasopressor, Methoxamine

## Abstract

**Background:**

α-receptor agonists have been reported to be safe and effective for treating or preventing spinal-induced hypotension during cesarean delivery. As a pure α_1_ adrenergic agonist, methoxamine has potential advantages of reducing myocardial oxygen consumption and protecting the heart in obstetric patients compared to phenylephrine. The aim of this study was to determine the optimal prophylactic methoxamine infusion dose that would be effective for preventing spinal-induced hypotension in 50% (ED_50_) and 95% (ED_95_) of parturients.

**Methods:**

Eighty parturients with a singleton pregnancy scheduled for elective cesarean delivery were randomly allocated to receive prophylactic methoxamine infusion at one of four different fixed-rates: 1 μg/kg/min (group M1), 2 μg/kg/min (group M2), 3 μg/kg/min (group M3), or 4 μg/kg/min (group M4). An adequate response was defined as absence of hypotension (maternal SBP < 80% of baseline or SBP < 90 mmHg). The values for ED_50_ and ED_95_ of prophylactic methoxamine infusion were determined by probit regression model. The outcomes of maternal hemodynamics and fetal status were compared among the groups.

**Results:**

The calculated ED_50_ and ED_95_ (95% confidence interval) of prophylactic methoxamine infusion dose were 2.178 (95% CI 1.564 to 2.680) μg/kg/min and 4.821 (95% CI 3.951 to 7.017) μg/kg/min, respectively. The incidence of hypotension decreased with increasing methoxamine infusion dose (15/20, 11/20, 7/20 and 2/20 in group M1, M2, M3 and M4 respectively, *P* <  0.001). 1-min Apgar scores and umbilical arterial PaO2 were lower but umbilical arterial PaCO2 was higher in Group M1. No difference was found in the other incidence of adverse effects and neonatal outcomes among groups.

**Conclusions:**

Under the conditions of this study, when prophylactic methoxamine infusion was given at a fixed-rate based on body weight for preventing spinal-induced hypotension in obstetric patients, the values for ED_50_ and ED_95_ were 2.178 μg/kg/min and 4.821 μg/kg/min respectively.

**Clinical trial registration:**

Chinese Clinical Trial Registry (ChiCTR), registry number of clinical trial: ChiCTR-1,800,018,988, date of registration: October 20, 2018.

## Background

Hypotension is a common complication caused by spinal anesthesia or combined spinal-epidural anesthesia for cesarean delivery [1], affecting up to 80% of patients if prophylactic vasopressors are not administered [2]. Both maternal and fetal/neonatal adverse effects are mostly correlated with severity and duration of post-spinal hypotension [3]. At present, the treatment of spinal-induced hypotension in obstetric patients relies on the use of vasopressors and intravenous crystalloid co-hydration; so the choice of vasopressors and the method of administration in obstetric patients are critical [4].. It is accepted that α-agonist drugs are the most appropriate agents to treat or prevent spinal-induced hypotension. Among all vasopressors, phenylephrine now is recommended as a first-line drug due to the amount of supporting data [4–6]. As another α-agonist drug, we think methoxamine is also suitable for obstetric patients.

Because the distributions of α_1_-adrenergic receptors on the surface of blood vessels are different in each part of human body, the organ’s responses to vasopressors are also different [7, 8]. The subtypes of α_1_-adrenergic receptors (α_1−_AR) are α_1A,_ α_1B_ and α_1D_; α_1A_ and α_1B_ are mainly distributed in peripheral blood vessels, while α_1D_ is predominant and functional in human epicardial coronary arteries [9]. Phenylephrine can activate all subtypes of α_1_-adrenergic receptors (α_1A,_ α_1B_ and α_1D_), whereas as a highly selective α_1_ adrenergic agonist, methoxamine activate only α_1A_ and α_1B_ [10–12]. Methoxamine has in theory less effect on myocardial contractility and consequently resulting in lower myocardial oxygen consumption when compared to phenylephrine. The heart protective effect of methoxamine may benefit for patients with coronary artery disease, but may not be necessary for healthy patients. Previous studies have suggested that methoxamine administered by continuous infusion is safe and effective for maintenance of hemodynamics [13, 14]. Methoxamine intermittent boluses or infusion have also been reported to be used in obstetric patients for prevention of spinal-induced hypotension [15, 16]. It has been well accepted that prophylactic infusion of a vasopressor is better than intermittent bolus use to prevent spinal-induced hypotension during cesarean delivery [3]. However, no previous studies about optimal doses for prophylactic methoxamine infusions have now been reported.

In the present study, we investigated different doses of prophylactic methoxamine infusion for preventing spinal-induced hypotension in obstetric patients. The aim of this study was to determine the optimal dose of prophylactic methoxamine infusion that would be effective for preventing hypotension in 50% (ED_50_) and 95% (ED_95_) of patients; secondary outcomes included maternal symptoms, haemodynamic changes and neonatal blood gas values.

## Methods

### Ethics and patients

After obtaining approval by the ethical review board of the Women’s Hospital, School of Medicine, Zhejiang University (approval number 20180095), and the registration in a Chinese Clinical Trial Registry (ChiCTR) (registration number ChiCTR1800018988; principle investigator: Fu Feng, M.D.; date of registration: October 20, 2018), we conducted a randomized, double-blinded dose-response study. Written informed consent was obtained from all subjects participating in the trial. This study adheres to CONSORT guideline.

Eighty parturients, American Society of Anesthesiologists physical status (ASA ≤ 2), with a singleton pregnancy scheduled for elective cesarean delivery were recruited. Patients were excluded if they had pre-existing or pregnancy-induced hypertension, diabetes mellitus, known cardiovascular or cerebrovascular disease, fetal abnormality, or contraindication to spinal anesthesia. The patients, whose body mass index > 30 kg/m^2^, height < 150 cm or > 170 cm, gestational age < 36 weeks were also excluded from the study.

### Grouping

Patients were randomly assigned into 4 groups based on computer-generated random number sheet. The patient in different group received one of four different fixed-rate prophylactic methoxamine infusion regimens as follows: 1 μg/kg/min (group M1), 2 μg/kg/min (group M2), 3 μg/kg/min (group M3), or 4 μg/kg/min (group M4). The range of infusion doses was based on the drug instruction, the consensus of Chinese experts in clinical anesthesiology and the previously published descriptions [13–15]. To maintain blinding, the solutions of methoxamine were prepared in identical 50-mL syringes by a co-investigator (Qian J P), who was not involved in data collection or clinical care of the patients. With the infusion rate predetermined to be set to 50 ml/h, the appropriate concentration of methoxamine was prepared by diluting to a 50 ml mixture with saline for each group. Then the milligram amounts of methoxamine were shown as follow: group M1, weight (kg) × 0.06; group M2, weight (kg) × 0.12; group M3, weight (kg) × 0.18; group M4, weight (kg) × 0.24.

### General management

Patients were subjected to 8 h food deprivation and 2 h water deprivation before surgery, without preoperative treatment. After arrival in the operating room, patients were allowed to rest for several minutes before measurements were taken. Standard noninvasive monitoring was applied, including non-invasive BP, pulse oximetry, and electrocardiography. The baseline systolic blood pressure (SBP) and maternal heart rate (HR) were determined from the mean of three readings that fell within 10% of each other, taken at least one minute apart. An IV line was established with an 18-gauge IV cannula in the forearm, and an infusion of lactated Ringer’s (LR) solution was started at a minimal rate to keep the vein open.

### Anesthesia procedures

Combined spinal-epidural anesthesia was performed with patients in the left lateral position. Epidural anesthesia was performed at the L1–2; 5 ml of saline was injected into the epidural space before insertion of the epidural catheter; while spinal anesthesia (midline puncture) was performed at L3-L4 or L4-L5.We injected the mixed intrathecal solution (1.5 ml of 1% hyperbaric Ropivacaine + 1.5 ml of 10% dextrose) into the subarachnoid space at the rate of 1 ml per 10 s. The 15 mg dose of hyperbaric ropivacaine was chosen because it is close to the ED_95_ for cesarean section in Chinese parturients reported by Chen X Z et al. [17] Immediately after the injection of the intrathecal medication, infusion of study drug of methoxamine was started at dosages of 1, 2, 3, and 4 μg·kg^− 1^·min^− 1^ (50 ml/h) respectively. Simultaneously, a fluid coload with 15 ml/kg of lactated Ringer’s solution over 20 to 30 min was started. Patients were then positioned supine with left uterine displacement and oxygen 5 L/min was administered via a facemask. The sensory block level of anesthesia, assessed by loss of pin prick discrimination, was recorded 5 and 15 min after induction of spinal anesthesia. The tested upper sensitivity block to T6 was considered adequate for surgery. However, the decision to allow surgery to start was based on clinical judgment of the attending anesthesiologist. In this study, we didn’t use the epidural catheter dose during the study period. If any patient received an epidural catheter dose, then they would have been excluded from the study.

### Monitoring and interventions

As per our standard practice, SBP, heart rate, and pulse oximetry were assessed every minute, commencing immediately after intrathecal injection until delivery and subsequently at 5-min intervals. Hypotension was defined as a decrease in SBP less than 80% of baseline value or SBP < 90 mmHg. If 90 mmHg ≤ SBP < 100 mmHg, it was treated with rapid infusion of lactated Ringer’s solution; if the SBP was < 90 mmHg, it was treated with a bolus of 3 mg IV methoxamine as rescue medication. Administration of a rescue bolus was repeated at 1-min intervals if hypotension persisted. We didn’t stop the treatment until hypotension improves or tends to improve [18, 19]. Reactive hypertension, defined as an increase in SBP to ≥20% above the baseline, was treated by stopping the infusion of methoxamine. Infusions were restarted only when the SBP decreased to < 120% of baseline. Bradycardia was defined as a heart rate < 50 beats/min; the bradycardia was managed by stopping the methoxamine infusion if no hypotension was present, and if hypotension was present during bradycardia then atropine 0.5 mg was given.

As our standard practice, the study was commenced immediately after intrathecal injection, until delivery of the baby. An effective methoxamine infusion dose was defined by the outcomes that patient did not have hypotension throughout the study period.

### Data collection

**Fig. 1:**
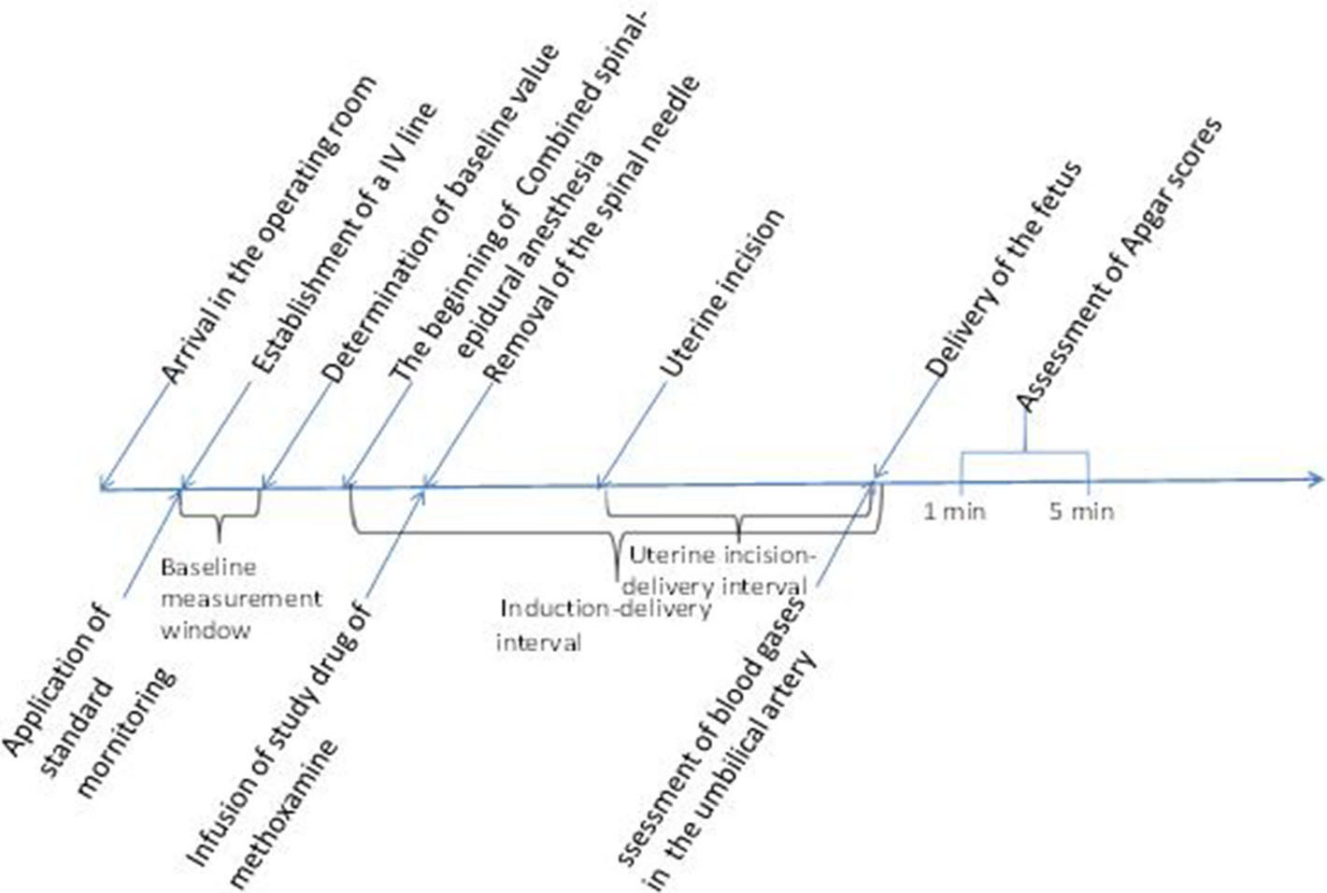
Study timeline: The period from the patient’s arrival in the operating room to the end of the study

Additional data collection included maternal demographics: age, weight, height etc. Time of fetal exposure to anesthesia and to surgical procedures were evaluated as induction-delivery interval (time from intrathecal injection to delivery of the fetus) and uterine incision-delivery interval (time from uterine incision to delivery of the fetus). Episodes of hypotension, hypertension, bradycardia, nausea and vomiting and shivering were recorded. Neonatal Apgar scores were measured at 1 min and 5 min. A segment of the umbilical cord was collected for assessment of blood gases in the umbilical artery. The timeline of the whole study [20] was shown as Fig. 1.

### Statistical analysis

Sample size estimation was determined by the Cochran-Armitage Test using PASS® (Version 11.0.7, NCSS, LLC, Kaysville, UT). According to and results from early preliminary data, the frequency of postspinal hypotension (70, 50, 30 and 10% respectively) in patients receiving one of the four infusion regimes same to that in the present study. The minimum effect size was 48patients in total (12 patients per group), which was found to have 90% power to detect a linear trend using a *Z* test with continuity correction and a significance level of 0.05. Allowing for possible dropouts, the sample size was increased to 80 patients (20 patients per group).

Data were expressed as mean and 95% confidence interval [mean (1.96 SD), 95% CI]. For numerical data, the Kolmogorov-Smirnov method was used to test for normal distribution, followed by one-way analysis of variance for normally distributed data and Kruskal-Wallis test for non-normally distributed data among the groups with post Bonferroni tests for pairwise comparisons. For nominal data, statistical analysis was performed by means of the Cochran-Armitage chi-square test for trend.

Dose–response data were analyzed with probit regression. Dose values were entered as x values, Y was the response as a percentage. The regression coefficient was obtained by regression analysis, then the ED_50_ and ED_95_ values were obtained from interpolation of the linear probit regression plot, and generation of the sigmoid dose-response plot was obtained secondarily. Serial changes in systolic blood pressure (SBP) of four groups are shown for the first 12 min after spinal anesthesia; they were analyzed using two-way ANOVA (include: different time points in the same group and different groups in the same time point). The correlation between duration of hypotension and 1-min Apgar score, duration of hypotension and umbilical arterial PaCO2 were analysed using Spearman’s rank correlation.

Analyses were performed using IBM SPSS Statistics for Windows version 22.0(IBM Corp, Armonk, NY) and GraphPad Prism version 5.0 (GraphPad Software Inc., San Diego, CA).All tests were two-tailed and *P*-value< 0.05 was considered statistically significant.

## Results

**Fig. 2:**
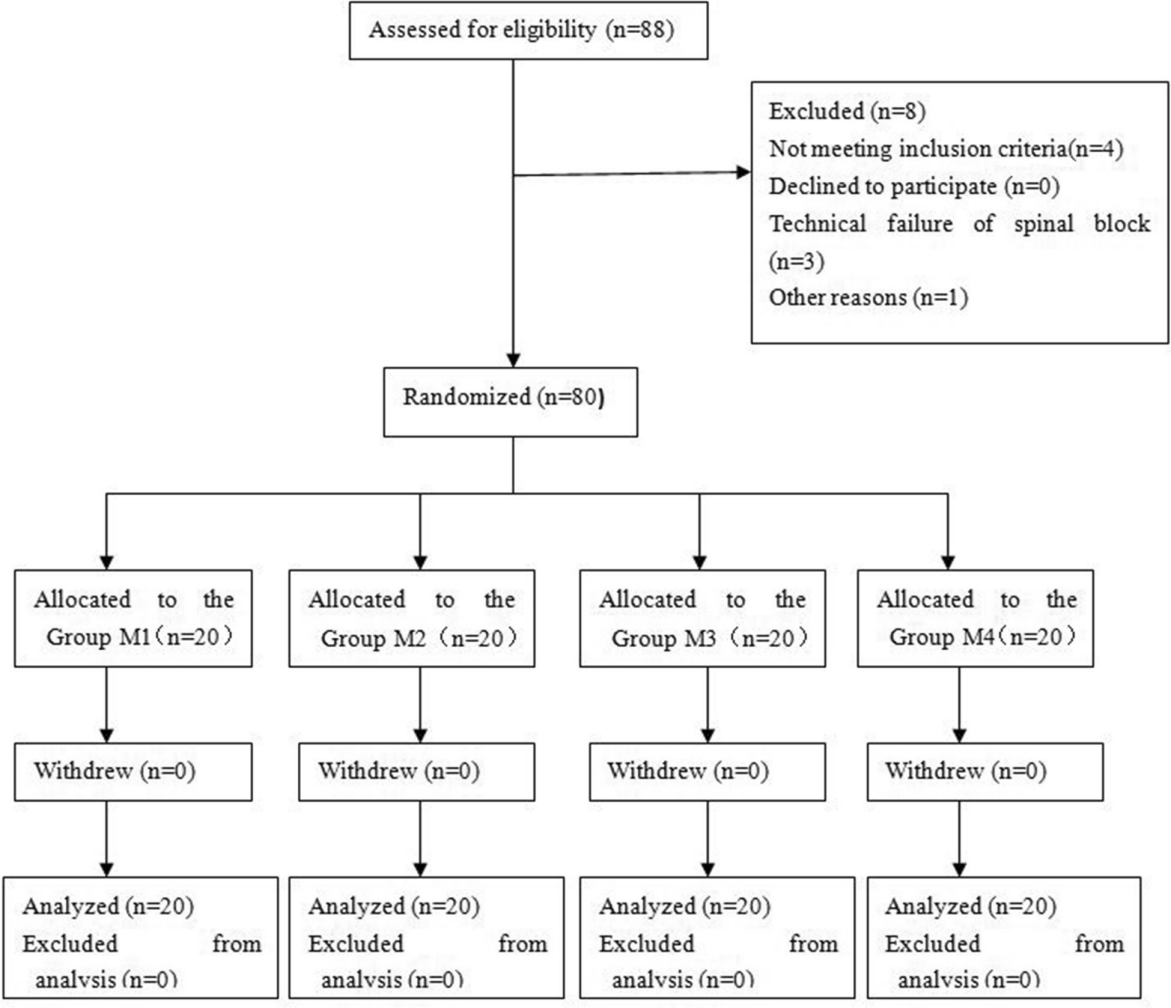
The Consolidated Standards of Reporting Trials flow diagram: Enrollment, randomization, and allocation of the study subjects

The trial flow diagram is shown in Fig. 2. A total of 88 parturients presenting for elective cesarean section were enrolled into the study; however, 4 patients did not meet the inclusion criteria, 3 patients had inadequate or failed spinal anesthesia and 1patient met too long U-D interval to analysis. Thus, the final analyses were confined to 80 parturients, with 20 subjects in each group. There were no significant differences among the groups in patient demographic characteristics, sensory block level, induction-delivery interval and uterine incision-delivery interval (Table 1.).

**Table 1:** Patient characteristic data, sensory block level and surgical times

	Group M1 (*n* = 20)	Group M2 (*n* = 20)	Group M3 (*n* = 20)	Group M4 (*n* = 20)	*P* value
Age (yr)	33.7 ± 5.3	34.3 ± 4.1	31.6 ± 4.0	32.8 ± 3.7	0.24
Weight (kg)	67.0 ± 6.7	66.6 ± 8.4	65.2 ± 7.1	67.0 ± 6.5	0.83
Height (cm)	161.0 ± 3.3	159.6 ± 5.2	158.7 ± 3.9	161.9 ± 4.7	0.09
BMI(kg m^−2^)	25.9 ± 2.5	26.1 ± 2.5	25.9 ± 2.5	25.4 ± 2.4	0.83
GA(wk)	38 (37 ~ 40)	38 (36 ~ 40)	38 (37 ~ 40)	38 (36 ~ 40)	0.62
Sensory block level (dermatome)	T4(T4 ~ 6)	T5(T4 ~ 6)	T4(T4 ~ 6)	T5(T4 ~ 6)	0.67
I-D interval(min)	13.7 ± 3.6	13.7 ± 2.8	12.1 ± 2.9	12.6 ± 2.7	0.26
U-D interval(s)	64.8 ± 11.5	61.9 ± 16.0	62.8 ± 20.7	64.5 ± 16.4	0.94

Data are presented as mean ± SD or median (range);

*GA* gestational age, *I-D* interval: induction-delivery interval, *U-D* interval: uterine incision-delivery interval

The incidence of intraoperative adverse events and physician interventions is presented as Table 2. The incidence of hypotension decreased with increasing methoxamine dose (75, 55, 35 and 10%; *p* <  0.0001); duration of hypotension also decreased along with group M1 to M4 (*p* <  0.0001). The incidence of reactive hypertension had an upward trend, but it was not statistically significant. There were no significant differences among the groups in the other incidences of adverse events including bradycardia, shivering and nausea and vomiting. The results showed the lower dose of methoxamine was used, the more rescue methoxamine bolus doses (*p* = 0.0001) and physician interventions (rescue vasopressor bolus, IV fluid bolus or atropine 0.5 mg etc.) were required (*p* = 0.0003).

**Table 2:** The incidence of intraoperative adverse events and physician interventions

	Group M1 (*n* = 20)	Group M2 (*n* = 20)	Group M3 (*n* = 20)	Group M4 (*n* = 20)	*P* value
Hypotension	15 (75%)	11 (55%)	7 (35%)	2 (10%)	< 0.0001
Reactive hypertension	0 (0%)	1 (5%)	2 (10%)	3 (15%)	0.058
Bradycardia	3 (15%)	3 (15%)	3 (15%)	0 (0%)	0.154
Nausea and vomiting	3 (15%)	1 (5%)	1 (5%)	2 (10%)	0.596
Shivering	3 (15%)	0 (0%)	1 (5%)	0 (0%)	0.067
Duration of hypotension for per patient (min)	2.0 ± 1.5	1.2 ± 1.3	0.7 ± 1.0	0.2 ± 0.6	< 0.0001
Number of patients who need rescue methoxamine bolus	14 (70%)	9 (45%)	7 (35%)	2 (10%)	0.0001
Number of patients who need physician interventions	15 (75%)	9 (45%)	9 (45%)	3 (15%)	0.0003

Data are presented as number (%), mean ± SD or median (range); Categorical data were analyzed using the Cochran-Armitage chi-square test for trend

Hypotension: SBP < 80% baseline or < 90 mmHg; Reactive hypertension: SBP > 120% of baseline value. Bradycardia: heart rate < 50 beats/min

**Fig. 3:**
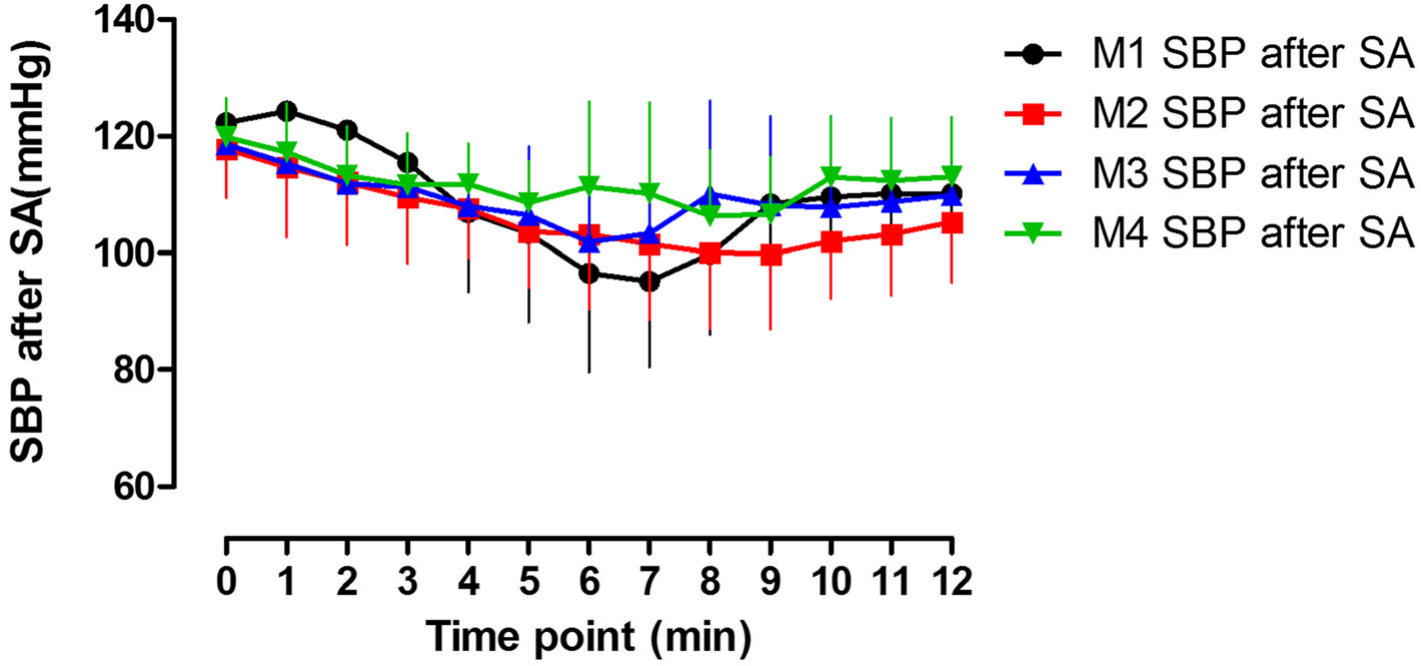
Serial changes in systolic blood pressure (SBP). Data are represented as mean (standard deviation) and are shown for the first 12 min after spinal anesthesia (SA) only. The data (mean SBP +/− SD.) over time was significantly different among groups (*P* < 0.001).

Serial changes in systolic blood pressure (SBP) for four groups in the first 12 min after spinal anesthesia (SA) are shown in Fig. 3. Analysis of SBP showed that changes in SBP were influenced significantly by both the dose of methoxamine and time (*P* <  0.05).

**Fig. 4:**
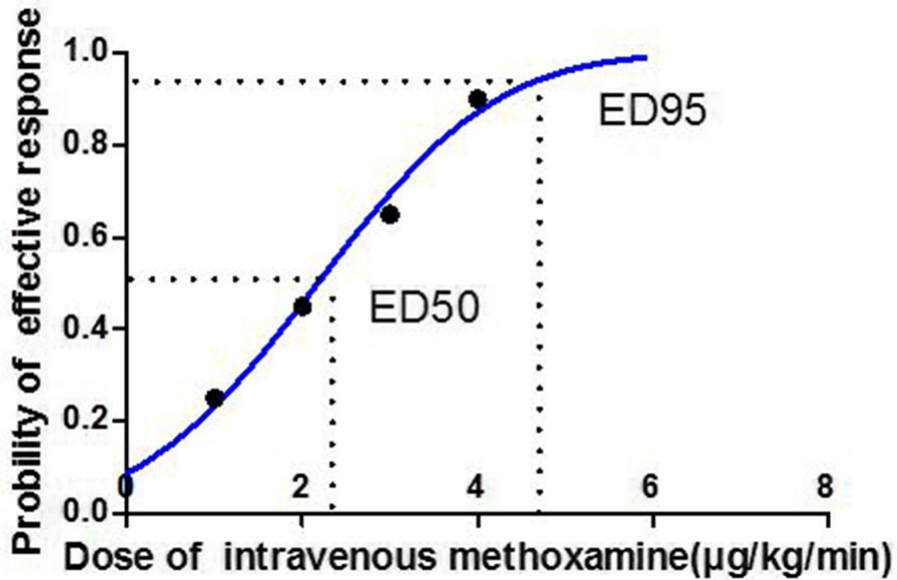
The dose-response curve of methoxamine infusions for preventing hypotension. The values for ED50 and ED95 calculated by Probit regression model were 2.178 (95% CI 1.564 to 2.680) μg/kg/min and 4.821 (95% CI 3.951 to 7.017) μg/kg/min respectively

The dose-response curve of methoxamine infusions for preventing spinal-induced hypotension is calculated by probit regression model shown in Fig. 4. The values for ED_50_ and ED_95_ were 2.178 (95% CI 1.564 to 2.680) μg/kg/min and 4.821 (95% CI 3.951 to 7.017) μg/kg/min, respectively.

**Fig. 5:**
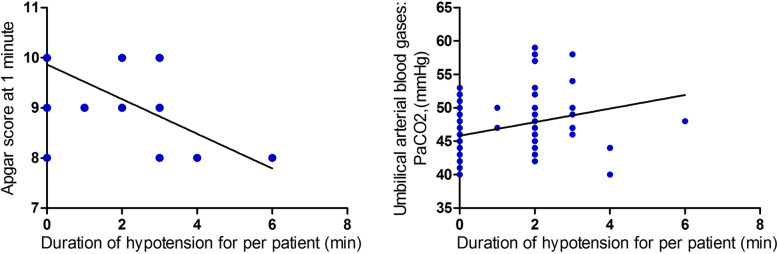
Correlation analysis between duration of hypotension and 1-min Apgar score, duration of hypotension and umbilical arterial PaCO2 respectively

Neonatal outcomes are presented in Table 3. 1-min Apgar scores were lower in group M1 [9 (8 ~ 10)] (*P* < 0.01). The outcomes of umbilical arterial blood gases showed PaO2 was lower but PaCO2 was higher in group M1 (*P* < 0.01). However, there were no differences in umbilical arterial blood PH and base excess among groups. Correlation analysis (Fig. 5.) showed a negative correlation between duration of hypotension and 1-min Apgar score (Spearman’s r: -0.74, *P* < 0.001), but a positive correlation between duration of hypotension and umbilical arterial PaCO2 (Spearman’s r: 0.33, *P* < 0.001).

**Table 3:** Neonatal outcomes

	Group M1 (*n* = 20)	Group M2 (*n* = 20)	Group M3 (*n* = 20)	Group M4 (*n* = 20)	*P* value
Apgar score at 1 min	9 (8 ~ 10)	9.5 (8 ~ 10)	10 (9 ~ 10)	10 (9 ~ 10)	0.008
Apgar score at 5 min	10 (10 ~ 10)	10 (10 ~ 10)	10 (10 ~ 10)	10 (10 ~ 10)	
Umbilical arterial blood gases:					
pH	7.27 ± 0.03	7.28 ± 0.02	7.27 ± 0.03	7.28 ± 0.02	0.64
PaO2(mmHg)	17.5 ± 4.2	21.9 ± 5.1	21.3 ± 4.4	21.7 ± 3.5	0.005
PaCO2,(mmHg)	49.0 ± 4.2	46.2 ± 4.0	47.7 ± 4.8	44.7 ± 3.3	0.008
Base excess(mM)	−1.2 ± 0.8	−1.1 ± 0.7	−1.1 ± 0.7	−1.6 ± 0.8	0.08

Data are presented as mean ± SD or median (range)

## Discussion

In this prospective, randomized double-blinded study, the dose–response relationships for prophylactic methoxamine infusion in patients undergoing combined spinal-epidural anesthesia for elective cesarean delivery were determined. The calculated ED_50_ and ED_95_ (95% confidence interval) for an effective prophylactic methoxamine infusion dose were 2.178 (95% CI 1.564 to 2.680) μg/kg/min and 4.821 (95% CI 3.951 to 7.017) μg/kg/min, respectively. This result may serve as a reference to guide for clinical infusion, but caution must be exercised because the calculated value of ED95 is above the maximum value tested.

In the present study, a probit regression model was used to determine values for ED_50_ and ED_95._ With this type of analysis, these values represent that the infusion dose of methoxamine would be effective for preventing hypotension in 50% (ED_50_) and 95% (ED_95_) of patients. According to these, the term *EDx* refers to the dose those results in an effective response in *X*% of patients [6]. The dose-response studies of phenylephrine infusion have been reported many times, but this is the first time for us to investigate optimal methoxamine infusion dose based on patient’s body weight by this means.

As we all know, the focus of how to prevent or treat spinal-induced hypotension has shifted to vasopressor drugs [21]. After decades of research, despite phenylephrine is currently considered a first-line vasopressor for prevention and treatment of spinal-induced hypotension during cesarean delivery, there are many other choices [22–24]. It is reported that norepinephrine and other alpha-adrenergic agonists have been chosen for management of spinal-induced hypotension during cesarean section. Methoxamine and phenylephrine have similar effects on vascular resistance, but methoxamine has relatively long duration of action and less potent [7]. What’s more, methoxamine has the potential advantage of reducing myocardial ischemia and protecting the heart, and tachyphylaxis has seldom been observed in methoxamine compared to other vasopressors [25]. Therefore, methoxamine is often used to maintain stable hemodynamics and suitable for elderly patients. However, the use of methoxamine in obstetrics was rare due to its potential adverse effects in obstetric patients. Caution must be exercised if methoxamine is used in the obstetric patients because methoxamine has tendency to produce uterine hypertonia which may result in fetal distress and it could interact with oxytocic drugs, subsequently result in postpartum hypertension [26]. In addition, the effect of methoxamine on uteroplacental flow is also controversial. Wright et al. demonstrated that methoxamine was associated with significant increase in uteroplacental resistance and flow [15], whereas Mintzer et al. showed methoxamine had no effect on uterine blood flow and the fetal arterial perfusion pressure [27]. Because these side effects of methoxamine in obstetrics are potential and controversial, we think it is also safe for obstetric patients. But there are insufficient supporting studies, so more data should be required before the use of methoxamine can be recommended in routine clinical practice for obstetric patients.

Several studies about phenylephrine showed that continuous infusion was superior to an IV bolus at preventing hypotension, nausea and vomiting and other intraoperative adverse events [5, 28, 29]. Similarly, a single intramuscular or intravenous injection for methoxamine has also been shown to have a slow onset and to cause unstable hemodynamics [8, 14]. In a previous study, they found that continuous intravenous infusion of methoxamine (2 μg/kg/min) can provide safe and effective maintenance of hemodynamics under epidural anesthesia during hip-joint replacement surgery in elderly patients [13]. However, now we found no other studies about optimal dose of prophylactic fixed-rate methoxamine for obstetric patients. Similar to the study by Xiao F et al. [30], we studied four prophylactic fixed rates for methoxamine infusion based on patient body weight during cesarean delivery under combined spinal-epidural anesthesia. Meanwhile the incidences of hypotension, hypertension and the number of physician interventions required were compared among 4 groups. Within a certain weight range (e.g. 60 kg to 80 kg), a fix-rate infusion dose with weight based is more accurate than that regardless of weight [31]. So, the infusion approach in this study might avoid under dosing in some patients and overdosing in the other patients.

In this study, we found greater hemodynamic stability in the higher dose of methoxamine fixed rate infusion (4 μg/kg/min). In fact, the incidence of hypotension decreased with the increase in dose of methoxamine. Correspondingly, the number of patients who required physician intervention was lower in patients who received the higher infusion dose in group M4. Unlike previous studies, the incidence of reactive hypertension was not high in our study. This result may be related to the mild effect of methoxamine on raising blood pressure [16]. Our study period (the period of continuous infusion of methoxamine) was not too long, which may also lead to the outcome of lower incidence of reactive hypertension.

Spinal-induced hypotension and the use of methoxamine can both cause bradycardia. This study showed there were no significant differences in bradycardia among the groups. This change in heart rate represents differences of the regulation of the autonomic nervous system among pregnant patients in response to spinal anesthesia [18]. Now more and more studies demonstrated heart rate variability can reflect the activity of the autonomic nervous system**,** and may be a tool to guide prophylactic therapy of patients at high risk for hypotension after spinal anesthesia. But this is beyond the scope of this article and requires further research.

Hypotension is believed to be the important etiological factor for intraoperative nausea and vomiting. Prophylactic methoxamine infusion regimens can also reduce the incidence of hypotension-induced nausea and vomiting [32]. There were no differences in the incidence of nausea and vomiting among the groups in our study, and our study was not powered to detect a significant association between methoxamine infusion dose and the incidence of nausea and vomiting too.

In our study, neonatal outcomes mainly included Apgar scores and umbilical arterial blood gases analysis. We found that 1-min Apgar scores and umbilical arterial PaO2 were lower, but umbilical arterial PaCO2 was higher in group M1. Some previous studies have mentioned duration of hypotension may be more important than severity of hypotension [3]. A transient decrease in blood pressure did not affect neonatal Apgar scores and the outcomes of umbilical arterial blood gases, whereas more than 4 min had a significant effect [3, 26]. We suspected that the duration of hypotension might be associated with neonatal outcomes. Our study showed a negative correlation between duration of hypotension and 1-min Apgar score, but a positive correlation between duration of hypotension and umbilical arterial PaCO2.The results meant that long duration of hypotension may reduce oxygen supply to the fetus and increase fetal carbon dioxide production, leading to lower PaO2 and higher PaCO2 [33]. We did not find Apgar scores below 7 in this study, which meant that the newborns were still within compensation range.

The present study has a number of limitations. First, we collected hemodynamic data for only the first 12 min at 1-min intervals in this study. So it is possible for us to miss hemodynamic changes in later periods that may affect our final outcomes. Second, the hemodynamic data in our study mainly included only maternal SBP and HR; we did not measure other hemodynamic variables such as maternal cardiac output. Third, the study was limited in the healthy pregnant patients undergoing elective cesarean delivery; further research must focus on the high- risk parturients. Finally, for descriptive purposes we included a large number of secondary outcomes which were not the primary objective of this study. In order to reduce the impact of possible false positive conclusions, most of the secondary outcomes should be considered as exploratory only.

## Conclusion

In conclusion, the present study demonstrated that the ED_50_ and ED_95_ of prophylactic methoxamine infusion for preventing spinal-induced hypotension in obstetric patients were 2.178 (95% CI 1.564 to 2.680) μg/kg/min and 4.821 (95% CI 3.951 to 7.017) μg/kg/min, respectively. Moreover, it is seemed that the higher dose of methoxamine administered as fixed rate, the more maternal hemodynamic stability and the better neonatal outcomes might be got. In order to confirm these issues, further studies are needed to investigate the application of methoxamine in obstetrics.

## Data Availability

The data that support the findings of this study in form of Excel files are available from the corresponding author.

## Abbreviations

ASA: American Society of Anesthesiologists physical status
SBP: Systolic blood pressure
HR: Maternal heart rate
LR: Lactated Ringer’s solution
SA: Spinal anesthesia
GA: Gestational age
I-D interval: Induction-delivery interval
U-D interval: Uterine incision-delivery interval
